# PIM Kinases as Potential Biomarkers and Therapeutic Targets in Inflammatory Arthritides

**DOI:** 10.3390/ijms25063123

**Published:** 2024-03-08

**Authors:** Elisa Assirelli, Jacopo Ciaffi, Valentina Scorcu, Susanna Naldi, Veronica Brusi, Luana Mancarella, Lucia Lisi, Federica Pignatti, Francesco Ursini, Simona Neri

**Affiliations:** 1 Medicine and Rheumatology Unit, IRCCS Istituto Ortopedico Rizzoli, 40136 Bologna, Italy; elisa.assirelli@ior.it (E.A.); jacopo.ciaffi@ior.it (J.C.); valentina.scorcu@studio.unibo.it (V.S.); susanna.naldi@ior.it (S.N.); veronica.brusi@ior.it (V.B.); luana.mancarella@ior.it (L.M.); lucia.lisi@ior.it (L.L.); federica.pignatti@ior.it (F.P.); simona.neri@ior.it (S.N.); 2 Department of Biomedical and Neuromotor Sciences (DIBINEM), Alma Mater Studiorum University of Bologna, 40126 Bologna, Italy

**Keywords:** rheumatoid arthritis, axial spondyloarthritis, psoriatic arthritis, PIM kinases

## Abstract

The Proviral Integration site for the Moloney murine leukemia virus (PIM)-1 kinase and its family members (PIM-2 and PIM-3) regulate several cellular functions including survival, proliferation, and apoptosis. Recent studies showed their involvement in the pathogenesis of rheumatoid arthritis RA, while no studies are available on psoriatic arthritis (PsA) and axial spondyloarthritis (axSpA). The main objective of this study is to assess the expression of PIM kinases in inflammatory arthritides, their correlation with proinflammatory cytokines, and their variation after treatment with biologic disease-modifying anti-rheumatic drugs or JAK inhibitors. We evaluated PIM-1, -2, and -3 expression at the gene and protein level, respectively, in the peripheral blood mononuclear cells and serum of patients with RA, PsA, axSpA, and healthy individuals (CTR). All the samples showed expression of PIM-1, -2, and -3 kinases both at the gene and protein level. PIM-1 was the most expressed protein, PIM-3 the least. PIM kinase levels differed between controls and disease groups, with reduced PIM-1 protein and increased PIM-3 protein in all disease samples compared to controls. No difference was found in the expression of these molecules between the three different pathologies. PIM levels were not modified after 6 months of therapy. In conclusion, our preliminary data suggest a deregulation of the PIM pathway in inflammatory arthritides. In-depth studies on the role of PIM kinases in this field are warranted.

## 1. Introduction

The Proviral Integration site for the Moloney murine leukemia virus (PIM) kinases are a family of protein kinases widely studied in the oncological field, given their importance in the development and progression of numerous types of cancer [1,2]. PIM kinases play a crucial role in controlling cell proliferation, cell cycle regulation, apoptosis, cell migration, and several other biological processes. Recently, their possible involvement has also been highlighted in several non-malignant diseases, including some rheumatological disorders [3,4].

PIM kinases are part of the CAMK (calcium/calmoduline-dependent kinases) subclass of serine/treonin kinases and were originally identified as a preferred site of the proviral insertion of the Moloney murine leukemia virus (MoMuLV) [5,6]. This family of kinases is composed of three different isoforms, encoded by the homonyms proto-oncogenes PIM-1, PIM-2, and PIM-3, which show a high homology of DNA and amino acid sequences [7,8]. Knockout mice for a single isoform do not present altered characteristics, and this implies a potential redundant function of the members of the PIM kinase family, which causes the deficit of one of them to be vicariate by the presence of the others. In knockout mice, for all three isoforms, no significant phenotypical alterations were observed, except for a reduction in the body size and mild alterations in the pathways regulating growth factors and T lymphocyte proliferation. Unlike other proteins, they have no regulatory domain and are constitutively active [9]. PIM kinases are ubiquitously expressed and are over-expressed in many neoplasms. Transcription of these proteins can be induced by various growth factors, pro-inflammatory cytokines, and mitogenic stimuli, which act through different signal transmission paths. The main molecules involved include GM-CSF, G-CSF, IFN-γ and IL-2, -3, -6, -7, -12, and -15. For most of these molecules, signal transduction occurs through the JAK-STAT pathway [7]. There is positive feedback between PIM kinases and the JAK/STAT pathway: PIMs induce the expression of certain cytokines, which, in turn, activate this transduction pathway [10]. At the same time, there may be negative feedback between PIM kinases and the suppressor of cytokine signaling (SOCS) [11]. The expression of PIM has been analyzed in several contexts, revealing potential participation in inflammatory diseases such as ulcerative colitis and cancers. The downward molecules of PIM kinases are involved in numerous biological and potentially pro-tumorigenic processes [7,12]. The role of these proteins in non-cancer cells is not yet fully clarified; however, their ability to regulate many cellular functions has been highlighted [13]. As for the involvement of PIMs in the immune response and inflammation, PIM kinases stimulate the survival of T and B lymphocytes, induce the differentiation of T lymphocytes in the different subtypes Th1 and Th2, activate the natural killer cells, and regulate the immunosuppressive function of Treg cells [14]. IL-6, IL-17, IL-1β, TNF-α, and IFN-γ are among the cytokines mainly involved in these functions [3,4,15,16].

It has been attributed a role for PIM kinases in various diseases characterized by inflammation and deregulation of the immune system [17,18]. Autoimmune disorders are pathological states brought on by an abnormal and protracted immune response. Only a few studies, mainly focused on rheumatoid arthritis (RA), have investigated the possible role of PIM in inflammatory arthritides. No studies are available on axial or peripheral spondyloarthritis. RA is the most common form of inflammatory arthritis where autoantigen-specific T cells and antibodies play crucial roles. RA is a chronic disease affecting about 0.5% of the adult population, and 30–40% of cases do not respond adequately to first-line treatment. On the other hand, psoriatic arthritis (PsA) and axial spondyloarthritis (axSpA) are inflammatory disorders characterized by innate immune system activation in the absence of autoantigen-specific T cells or autoantibodies. PsA is associated with the presence of skin psoriasis affecting about 0.2% of the population; axSpA is characterized by inflammation of sacroiliac joints and the spine, with possible peripheral manifestations [19,20]. First-line treatment of patients with peripheral arthritis mainly relies on the use of conventional synthetic disease-modifying anti-rheumatic drugs (cDMARDs), with the possibility to introduce biologic DMARDs (bDMARDs) or Janus Kinase Inhibitors (JAKi) in inadequate responders [21,22]. In patients with axSpA, bDMARDS or JAKi should be considered after failure of non-steroidal anti-inflammatory drugs (NSAIDs) [23].

One of the earliest studies concerning PIM kinases and inflammation was that of Yang J. et al. [15], which showed that PIM-2 influences the expression of IL-6, a cytokine that plays a central role in chronic inflammation and RA pathogenesis. A correlation between NF-kB, one of the central molecules in the regulation of inflammation in RA, and PIM kinases was studied by the group of Yin G. et al. [24]. The team of Anderson et al. [25] observed that PIM-1 expression was increased in circulating CD4+ T lymphocytes in patients with untreated early RA, with significant differences compared to healthy controls. This increase was mediated by the STAT3 pathway, which in turn was triggered by IL-6 [26,27]. The involvement of PIM kinases in RA pathogenesis was also suggested by the observation of higher PIM-1 expression in different cell types of the synovia of early RA patients compared to controls. In addition, inhibition of PIM-1 with PIM kinase selective molecules or pan-PIM inhibitors caused a decrease in the activation and proliferation of CD4+ T lymphocytes, a reduction in the production of pro-inflammatory cytokines, in particular IFN-γ and IL-17, and an increase in Treg lymphocytes [28]. Finally, in mice with induced early RA patterns and treated with the same inhibitors, a reduction in the severity of arthritis with decreased cartilage destruction was observed, suggesting PIM kinase inhibitors as possible therapeutic molecules in early RA patients [26,27]. This was confirmed by Ha Y.-J. et al. [16] in synovial cells of RA patients showing higher levels of PIM-1 than controls. After PIM-1 silencing, a reduction in viability, proliferation, and migration of synoviocytes, as well as a decrease in the production of metalloproteases, was described.

Growing evidence of PIM kinases’ involvement in immune-mediated and inflammatory disorders points to their potential application as therapeutic targets in these diseases. PIM kinase regulates T and B cell function, which is implicated in important inflammatory and autoimmune processes. The JAK/STAT pathway, a crucial signal transduction system that is dysregulated in autoimmune and inflammatory diseases, controls the expression of PIM kinases. JAK kinase inhibitors are marketed with caution due to possible serious adverse effects, despite their successful development for conditions like psoriasis, inflammatory bowel disease, and rheumatoid arthritis [29].

The central role of PIM kinases in the pathogenesis of tumors has led them to become a target widely studied in pharmacological research in the oncological field. Various PIM kinase inhibitors are described in the literature; however, there is no molecule available on the market at present, due to the long failure of clinical trials. Since the three PIM kinases have largely overlapping functions with each other, a pan-PIM inhibitor is preferable to a selective inhibitor for a specific PIM kinase. Most of the inhibitors synthesized so far are pan-PIM inhibitors. They are mainly classified into benzofurans, indols, oxadiazols, pirazines, pyrimidines, pirrols, quinolines, tiazolidines, triazols, and their derivatives. Some of the main ones, also used in the mentioned studies, are AZD1208 and SMI-4a [30]. Through PIM kinase inhibition, downstream cellular targeting of the JAK/STAT pathway may lessen these negative effects, enabling a safer therapeutic approach. In general terms, the synthesis and design of kinase inhibitors in silico involves the use of computational techniques to predict and optimize molecules that can effectively inhibit the activity of specific kinases. The selective process includes several steps that combine computational modeling and experimental validation (briefly: target selection; structure determination; virtual screening; lead optimization; assessment of absorption, distribution, metabolism, excretion, and toxicity profiles; validation; iterative optimization) to expedite the discovery and optimization of novel therapeutic agents [31,32].

We here evaluated the expression of PIM kinases in peripheral blood mononuclear cells (PBMC) and serum of RA, PsA, and axSpA patients compared to controls (CTR), correlated enzyme levels to the expression of inflammatory cytokines, and evaluated the effect of bDMARD and JAK inhibitors treatment.

## 2. Results

The expression of PIM kinases (PIM-1, PIM-2, and PIM-3) was analyzed in RA, PsA, and axSpA patient samples naïve to biologics or JAKi, some of which already received prior treatments with cDMARDs or corticosteroids. Therefore, before grouping samples, the possible effect of these treatments on baseline PIM levels has been assessed. Baseline levels (T0) of protein and mRNA for PIM-1, PIM-2, and PIM-3 in patients with RA, axSpA, and PsA who had not received any disease-modifying therapy were compared to patients who had previously received treatment with cDMARDs or corticosteroids. No statistical differences were observed both in gene and protein expression, even when patients were categorized according to their diagnosis (Mann–Witney U test NS). The only exception was the protein expression of PIM-3, where patients previously treated with cDMARDs or corticosteroids had significantly higher mean values than naive patients (*p* = 0.006).

Since there were no major differences in PIM expression between cDMARD-naïve and cDMARD-experienced patients, the analyses were conducted using the whole group of patients with inflammatory arthritides.

### 2.1. Relative Expression of PIM-1, PIM-2, and PIM-3 Kinases in Inflammatory Arthritides

To assess differences in the absolute expression of the three forms of kinases, serum protein levels were compared with each other in RA, axSpa, PsA, and controls.

**Figure 1:**
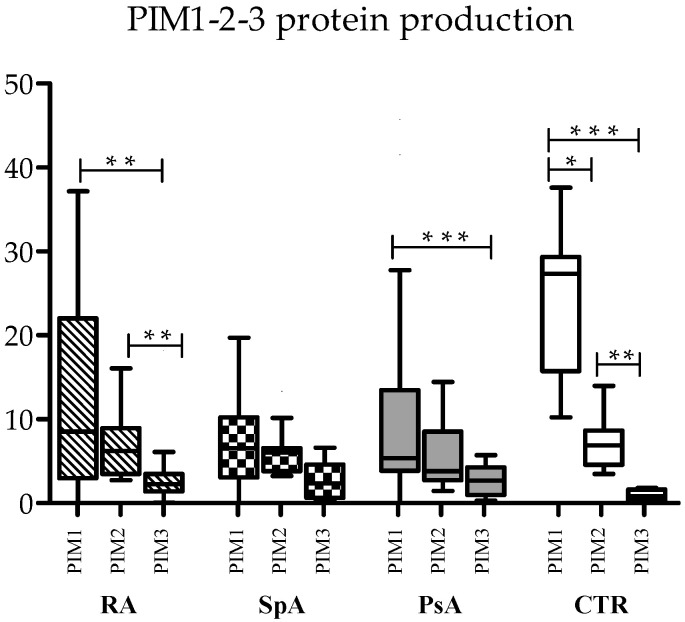
Relative expression of PIM-1, PIM-2, and PIM-3 kinases in inflammatory arthritides. PIM-1, PIM-2, and PIM-3 basal production in the serum of RA, axSpA, PsA, and CTR donors is shown. The Kruskal–Wallis test followed by Dunn’s post hoc test was used to compare the amount of the three kinases. Boxes represent 25th–75th percentiles, and horizontal lines represent medians; whiskers represent minimum and maximum values; * *p* ≤ 0.05; ** *p* ≤ 0.01; *** *p* < 0.001.

In control samples, PIM-1 was the most prevalent kinase (median 27.35 ng/μL), followed by PIM-2 (median 6.89 ng/μL) and PIM-3 (median 0.81 ng/μL), with statistically significant differences (Figure 1). This trend was maintained also in the three diseases, even if some differences lost statistical significance, mainly due to reduced PIM-1 levels in the three pathologies. In RA, both PIM-1 and PIM-2 were significantly more expressed than PIM-3, and in PsA, PIM-1 was more expressed than PIM-3 (Figure 1).

As concerning the relationship between the three kinases, PIM-1 expression was directly correlated with PIM-2 expression, both at the RNA and protein level in controls (Spearman’s correlation: R = 0.680, *p* = 0.015 for RNA; R = 0.762, *p* = 0.004 for proteins). This was also observed in RA (Spearman’s correlation: R = 0.524, *p* = 0.031 for RNA; R = 0.675, *p* = 0.001 for proteins) and PsA (Spearman’s correlation: R = 0.571, *p* = 0.013 for RNA; R = 0.752, *p* < 0.0001 for proteins), while in axSpA, only RNA levels were positively correlated. No correlation was found between proteins (Spearman’s correlation: R = 0.762, *p* = 0.037 for RNA; R = 0.423, *p* = ns for proteins).

All three PIM kinase proteins exhibited positive correlations in the control samples as well (Spearman’s correlation: PIM-1 vs. PIM-2, R = 0.762, *p* = 0.004; PIM-1 vs. PIM-3, R = 0.692, *p* = 0.013; PIM-2 vs. PIM-3 R = 0.678, *p* = 0.015), but this was not observed in any of the three diseases.

### 2.2. Basal Expression of PIM-1, PIM-2, and PIM-3 Kinases in RA, axSpA, and PsA Compared to Controls

**Figure 2:**
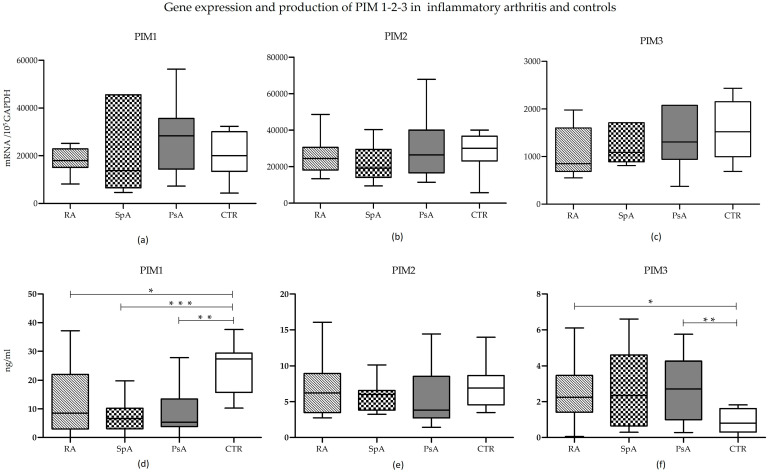
The PIM-1, PIM-2, and PIM-3 mRNA (upper panels) and protein (lower panels) expression in RA, axSpA, PsA, and controls (CTR). The expression levels of PIM-1 (**a**,**d**), PIM-2 (**b**,**e**), and PIM-3 (**c**,**f**) kinases in the four groups were compared using the Kruskal–Wallis test (RA vs. axSpA vs. PsA vs. CTR), followed by Dunn’s post hoc test. Boxes represent 25th–75th percentiles, and horizontal lines represent medians; whiskers represent minimum and maximum values, * *p* ≤ 0.05; ** *p* ≤ 0.01; *** *p* < 0.001.

Gene expression analysis at T0 showed no significant differences in mRNA levels of the three PIM kinases, neither within the RA, axSpA, and PsA groups nor between patient samples and controls (Figure 2, upper panels).

Conversely, the protein expression of the three molecules showed some differences (Figure 2d–f). In all three conditions, PIM-1 was produced to a significantly smaller extent than in controls (RA vs. CTR: *p* = 0.0273; axSpA vs. CTR: *p* = 0.003; PsA vs. CTR: *p* = 0.0023). Differences among individual diseases were not significant (Figure 2d). While not reaching statistical significance, PIM-1 protein tended to be highly expressed in RA compared to both PsA and axSpA, likely due to the presence of outliers.

PIM-2 protein expression showed no variations in the four groups (Figure 2e).

Finally, the expression of PIM-3 protein was similar in the three diseases but tended to be lower in controls (Figure 2f), reaching statistical significance in the case of RA and PsA (RA vs. CTR: *p* = 0.0176; PsA vs. CTR: *p* = 0.006).

### 2.3. Effect of Pharmacological Treatment on PIM-1, PIM-2, and PIM-3 Expression

**Figure 3:**
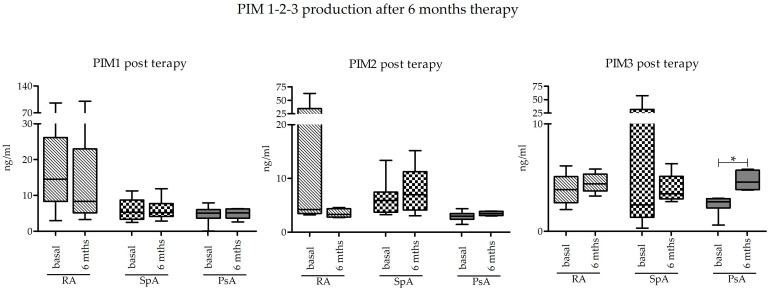
PIM-1, PIM-2, and PIM-3 production after 6 months of bDMARD or JAK inhibitor treatment in the 4 different groups of donors (RA, axSpA, PsA, and CTR). Comparisons were made by the Mann–Whitney U test. Boxes represent 25–75th percentiles, and horizontal lines represent medians; whiskers represent minimum and maximum values, * *p* ≤ 0.05.

Basal levels of PIM kinase expression at T0 were compared to those obtained after 6 months of treatment with bDMARD or JAK inhibitors (T6) in a subgroup of subjects. Looking at the data as a whole, there were no differences in the expression of PIM kinases after 6 months. The only significant difference was appreciated in the group of PsA as regards PIM-3 protein, which was indeed significantly increased (*p* = 0.0313) (Figure 3).

### 2.4. Relationship between PIM Kinases and Inflammatory Markers

A series of inflammatory molecules related to PIM kinase pathways (IL-17, IL1-β, IL-6, TNF-α, IFN-γ) were evaluated at the mRNA and protein level, respectively, in the PBMC and serum of RA, axSpA, PsA, and CTR samples. The mean ± SD levels of these markers in the four groups are shown in Supplementary Table S2.

Correlations between these markers (RNA and proteins) and PIM kinases (RNA and proteins) were analyzed to check for differences among pathologies and compared to controls (the correlation coefficients and statistical significance are available in Supplementary Table S3).

The analysis highlighted several differences between patients and controls. IL-17 protein was inversely correlated to PIM-1 protein levels only in PsA samples (*p* = 0.024). IL-17 RNA levels were also directly correlated to PIM-3 in controls (*p* = 0.022) and axSpA (*p* = 0.008) but inversely correlated to PIM-3 in RA (*p* = 0.029) and not correlated in PsA.

In controls, IL-1β positively correlated with PIM-1 both at the RNA (*p* = 0.007) and protein (*p* = 0.040) levels, whereas no correlations were found in RA, PsA, and axSpA. Again, in controls, IL-1β positively correlated with PIM-2 both at the RNA (*p* = 0.003) and protein (*p* = 0.011) level, while in RA samples, this occurred only for PIM-2 protein (*p* = 0.025), and no correlations were found in axSpA and PsA samples. Finally, IL-1β negatively correlated with PIM-3 protein in controls (*p* = 0.033) and in RA samples (*p* = 0.026).

IL6 showed correlations with PIM kinases in control samples and in any of the pathologies. In controls, IL-6 RNA levels positively correlated with PIM-1 (*p* = 0.024) and PIM-2 (*p* = 0.044) RNA levels; again, in controls, IL-6 protein levels positively correlated with PIM-2 (*p* = 0.044) protein levels.

TNF-α RNA positively correlated with PIM-1 (*p* = 0.004) and PIM-2 (*p* = 0.012) RNAs in control samples and with PIM-2 RNA in axSpA (*p* = 0.007). No correlations were found between PIM kinases and TNF-α proteins.

IFN-γ protein positively correlated with PIM-1 (*p* = 0.047), PIM-2 (*p* = 0.017), and PIM-3 (*p* = 0.020) proteins only in control samples, while in the three pathologies, only negative correlations or no correlations with PIM proteins were noticed (Table S3).

## 3. Discussion

The present work assessed the involvement of PIM kinases in the regulation of inflammation in chronic inflammatory arthropathies. We investigated PIM kinases not only to deepen their biological role, as yet unknown, but also to evaluate their potential as biomarkers. The expression level of these molecules has some interesting differences between the groups of subjects examined. Moreover, interesting data emerged from the comparison and correlation of PIM kinases among themselves and the correlations between their expression and the expression of a series of molecules connected to their pathway and/or involved in the inflammatory response.

No differences in gene expression emerged from the analysis of the three PIM kinases in the patient groups and in the control group. These observations suggest that the possible adjustment of PIM kinase expression is more likely to take place at the post-transcriptional level, thereby essentially modulating the production of protein rather than mRNA. At present, the post-transcriptional regulation mechanisms described in the literature are few. There are several factors that can increase or decrease the stability of these proteins, including the main protein phosphatase 2A (PP2A) and the heat shock proteins HSP70 and HSP90 [33,34]. Moreover, very often mRNA levels do not correlate with those of proteins, either because the kinetics of production by cells are not the same or because the regulatory mechanisms can act at different levels [7,14]. PIM-1 protein expression in the three groups of patients was significantly lower than in the control group. These data appear to contrast with the results produced by other studies describing an increased PIM-1 expression in RA patients [3,16]. This divergence can be mainly due to some methodological aspects. Firstly, previous studies have been conducted on selected cell subpopulations and, in particular, the expression of PIM kinases has been evaluated on circulating CD4+ T lymphocytes and on synovial cells. Our evaluations were completed in serum samples and on total PBMC RNA, thus measuring the protein production of different cell types including not only circulating CD4+ T lymphocytes but also other PBMCs and molecules released in the circulation by cells of the inflamed synovium or other tissues [25,26,27]. In addition, in previous studies, different laboratory techniques were used, specifically immunohistochemistry, immunofluorescence, and cell cytometry. Reflecting on the data that emerged from our study, the expression of PIM-1 could derive from negative feedback control, as described in the literature between PIM kinases and SOCS [12]. While the production of PIM-2 showed no significant differences between the various groups analyzed, the production of PIM-3 protein showed an opposite trend to that of PIM-1: the levels of PIM-3 were lower in controls than in the three pathologies, and in the case of RA and PsA, this difference was significant.

It must be considered that the members of the PIM kinase family have a redundant function; consequently, if PIM-1 levels decrease during inflammation in our experimental model, PIM-3 could be up-adjusted to compensate for the reduction in PIM-1 [9]. Another possible interpretation is that the overall balance of PIM-3 production by the various cell types is in favor of PIM-3. Based on a comparison of PIM kinase expression within each group, PIM-1 is the most abundant, while PIM-3 is the least expressed. Comparing these differences between the four groups, it appears that the mutual relationships between the levels of the three PIM kinases are greater in the controls, while, in the case of the three pathologies, they tend to decrease, and the medians become more similar to each other. This also supports the hypothesis that, in the presence of inflammation, a series of molecules occur in PIM kinases’ expression regulation. We then focused on the expression of PIM kinases after six months of therapy with bDMARD or JAK inhibitors in order to investigate whether immunosuppressive therapies modulate the expression of our molecules of interest. Combining all the diseases together, some trends could be observed. Specifically, there was a tendency to reduce the expression of PIM-1 from T0 to T6 and, on the contrary, a trend toward PIM-3 upregulation. We then analyzed data also after sample stratification according to the type of arthritis, and we found that PIM-3 was upregulated in PsA after six months of treatment. These comparisons, like most of the analyses carried out in this work, suffer from the limit of the reduced number of samples, which is probably one of the reasons that has made it difficult to reach statistical significance [5]. bDMARD and JAK inhibitors can cause a partial modulation of PIM kinase expression by acting on the pro-inflammatory cytokines related to PIM activity, such as the already mentioned IL-17, IL-1β, IL-6, TNF-α, and IFN-γ. Furthermore, it should be considered that JAK inhibitors act on the JAK/STAT pathway, which is one of those mainly involved in PIM expression. It would, therefore, be interesting to deepen the research both by extending the analysis to wider case series for a successful stratification according to the type of drug taken and by using PIM-specific kinase inhibitors. Correlations have been found between PIM and some pro-inflammatory molecules, such as IL-17, IL-1β, IL-6, TNF-α, and IFN-γ that, at the same time, play a crucial role in the pathophysiology of chronic inflammatory arthropathies [16]. In our case series, these molecules did not show significant differences among the analyzed groups, but they correlated with PIM expression. By correlating the expression of PIM kinases, both among themselves and to the expression of the analyzed cytokines, some interesting results were obtained. The levels of expression of PIM kinases appeared to be positively correlated with each other in controls, but these correlations were almost all lost in the three disease groups, suggesting the deregulation of the PIM kinase pathway in pathological samples, according to the observation that pathological samples showed reduced PIM-1 and increased PIM-3 levels compared to controls. This is also in line with the known redundant role of the members of this family of kinases, with an increase in PIM-3 corresponding to the PIM-1 decrease, suggesting a vicariant role of PIM-3 compared to the other members of the PIM kinase family. Even in this case, our data suggest a post-transcriptional rather than transcriptional modulation of expression [9]. As concerning PIM expression in relationship with pro-inflammatory cytokines, we again observed a different behavior in disease groups compared to controls. In the latter, PIM expression mostly positively correlated with inflammatory cytokine production, while these correlations were almost completely lost in the three groups of disease, again suggesting a deregulation of the PIM pathway. IL-17 was negatively correlated with PIM-1 and PIM-3 in RA, and PsA groups, which is in line with results from Buchacher et al. [35], who also observed a negative regulation of PIM-1 on IL-17 expression in vitro. As concerning IL-1β, IL-6, and TNF-α, positive correlations were observed mostly in the control group, while no correlations emerged in the disease groups. This could depend on the central role of synovial cells in the production of this biomarker in inflammatory arthritides that we have not investigated here [36]. Accordingly, IFN-γ, a central inflammatory molecule mainly produced by activated T cells, showed a direct correlation with all PIM kinases, again observed in controls and absent in the three disease groups [36]. The observed positive correlation of PIM-1 with TNF-α and IFN-γ is in accordance both with Maney et al. [3], showing reduced IFN-γ levels after the inhibition of PIM-1, and with Ha Y.-J. et al., showing a stimulating effect of TNF-α increase on PIM1 expression [16]. Numerous papers have demonstrated the function of PIM-2 in decreasing inflammation and restoring homeostasis. Specifically, this function of PIM-2 has been found in RA in the case of oxidative stress caused by lipid peroxidation and in atherosclerosis, a condition in which PIM-2 plays a protective role against inflammation [24,37]. Considering this evidence, we must note that PIM-2 expression is essentially steady in our experimental model. From a protein or gene perspective, this molecule does not differ significantly across the various diseases or between the arthropathies and the controls. Moreover, its protein expression remains unchanged following 6 months of treatment with bDMARD or JAK inhibitors. Considering the correlation of PIM-2 gene expression with pro-inflammatory cytokines, a positive association with IL-1β, IL-6, TNF-α, and IFN-γ is shown mainly in control samples, similar to what is described for PIM-1. Also, in this case, data are in line with those from the literature. Specifically, in Yang J. et al.’s study, a link was found between increased levels of IL-1β and TNF-α and increased PIM-2 expression, with the former stimulating the expression and activity of PIM kinase through the NF-kB pathway [38].

In this paper, we investigated some aspects that have not been considered in the literature so far. Actually, there is some evidence with respect to RA, while no data are available on other inflammatory arthritides. Here, we evaluated PIM expression in SpA and PsA compared to RA and to controls. Our data did not highlight significant differences among the three diseases, whereas they differed from controls. Another contribution of this study was to extend the analysis of gene and protein expression to PIM-3, which had so far never been evaluated in rheumatology, unlike PIM-1 and PIM-2 kinases. From these results, PIM-3 might be the member of the PIM kinase family, suggesting the most interesting developments. In fact, the study highlights how its expression appears to be the most involved in the analyzed diseases at the expense of PIM-1.

However, the results of this study should be interpreted in light of the limitations imposed by the sample size, which may have masked some differences that failed to reach statistical significance, particularly in the subgroups. Later studies, based on more cases, could allow for the identification of differences between specific groups of subjects. In fact, it must always be considered that the pathway of PIM kinases may be active only in certain groups of patients, as already suggested by other studies [3].

Furthermore, regarding the baseline samples, some of the patients may have taken other drugs, such as corticosteroids or anti-inflammatory treatments that also have immunosuppressive properties, as well as cDMARD, even if they had never received treatment with bDMARD or JAK inhibitors at the time of sampling. Similarly, due to the small number of included patients, we were unable to stratify or adjust our analysis taking into account comorbidities and ongoing non-rheumatological therapies. In conditions such as inflammatory arthritis, it is exceedingly challenging to obtain a sizable sample of patients who are not receiving any form of treatment. Baseline samples were performed a few days before starting the treatment with a bDMARD or a JAKi treatment.

The results of our study should be considered preliminary until not confirmed in larger samples. However, the presented observations could serve as a starting point for further research. Possible developments could be the evaluation of PIM kinase expression on specific populations of cells of the immune system and/or in synovial cells and the investigation of PIM kinase modulation after cellular stimulation in vitro. Moreover, further studies could evaluate the correlation between PIM kinase expression and clinical parameters such as disease activity, disease duration, demographics, and other characteristics of patients. The implications for clinical practice can be protean, but further considerations might appear speculative given the exploratory nature of our study. Theoretically, an in-depth analysis of how PIM kinase expression is modulated by each individual medication for RA, PsA, and axSpA might provide further insights into the pathogenetic role of these molecules and their relationship with disease trajectories. Furthermore, the limited sample size of our study precluded the possibility of clustering patients and exploring PIM kinases in specific disease subsets and/or other demographic/clinical characteristics. Our findings contribute relevant data to the currently limited knowledge about PIM kinases in inflammatory arthritides, but additional translational research on PIM inhibitors is warranted to hypothesize their application in patients with rheumatic diseases.

## 4. Materials and Methods

### 4.1. Patients and Samples

Peripheral blood samples from 23 RA patients (12 Female (F)/11 Male (M); mean age 61 ± 16 years), 12 axSpA patients (1 F/11 M; mean age 41 ± 15), 24 PsA patients (12 F/12 M; mean age 54.5 ± 14), along with 13 control subjects with no autoimmune or inflammatory diseases (11 F/2 M; mean age 59.5 ± 19) were obtained from the Rheumatology Biobank of the Istituto Ortopedico Rizzoli [39]. All patients participating in the Biobank project had provided written informed consent, and the study was approved by the CE-AVEC Ethical Committee (protocol N. 206/2023/Sper/IOR). For each patient, mononucleate cells and serum were obtained from peripheral blood with standard procedures [20]. Samples were recovered and analyzed at time T0, corresponding to patients naïve to therapies with bDMARDs and to JAKi, including those who have never received therapy before as well as those who have previously received cDMARDs (3 RA and 6 PsA) or corticosteroids (5 RA and 1 PsA) (Supplementary Table S1). A subgroup of these patients was also analyzed after 6 months bDMARD or JAKi therapy (T6).

### 4.2. Gene Expression Analysis

Total cellular RNA was obtained from mononuclear cells (0.5–2.0 × 10^6^) with Eurogold Trifast reagent (Euroclone), and reverse transcription was performed by random hexamer priming using the SuperScript VILO cDNA Synthesis kit (Life Technologies, New York, NY, USA), following the manufacturer’s instructions; mRNA expression was evaluated by semi-quantitative Real-Time RT-PCR in a Light Cycler Instrument (ROCHE Molecular Biochemicals, Mannheim, Germany) using the SYBR Premix Ex Taq (TAKARA Biomedicals; Tokyo, Japan) with the following protocol: 95 °C for 30 s, 40 cycles at 95 °C for 5 s, and 60 °C for 20 s. Primer sequences are reported in Table 1. Amplicon specificity was checked at each run by melting curve analysis. The Ct (Cycle threshold) values were determined for each sample. Relative mRNA expression was quantified with respect to the glyceraldehyde-3-phosphate dehydrogenase (GAPDH) housekeeping gene following the formula (1 + E) ΔCt, where E represents the reaction efficiency (approximated to 1 because >90% for all the transcripts) and ΔCt the difference between the GAPDH and the specific Ct for each sample. Data are expressed as mRNA copy number per 100,000 GAPDH copies.

### 4.3. Soluble Factor Quantification

PIM1, PIM2, and PIM3 concentrations in the serum were measured by FineTest (Wuhan, Hubei, China) commercial ELISA kits, according to the manufacturer’s instructions (PIM-1 protocol https://www.fn-test.com/product/eh0206/, (accessed on 8 January 2024); PIM-2 protocol https://www.fn-test.com/product/eh11135/, (accessed on 8 January 2024); PIM-3 protocol https://www.fn-test.com/product/eh2293/, (accessed on 8 January 2024).

Serum concentrations of IL1β, IL-17, IL-6, TNFα, IFNα, IL-10, visfatin, leptin, and resistin were simultaneously evaluated by a custom 9-plex Luminex Human Discovery Assay (Biotechne, Minneapolis, MN, USA). Conversely, serum adiponectin has been measured independently using a 1-plex Luminex Human Discovery Assay (Biotechne, Minneapolis, MN, USA) due to its significantly higher concentration than other analytes. The bead-based sandwich immunoassays were performed following the manufacturer’s instructions. The immunocomplexes formed on distinct beads were quantified with the BioPlex Protein Array System (Bio-Rad Laboratories). Version 6.0 of the Bio-Plex Manager program was used to analyze the data (Bio-Rad Laboratories, Hercules, CA, USA). Standard levels between 70% and 130% of the expected values were considered accurate and were used.

### 4.4. Statistical Analysis

Data were expressed as medians, interquartile ranges, minimum and maximum values, and means ± standard deviation (SD), as appropriate. Differences between independent samples were analyzed by the Mann–Whitney U test, whereas differences between paired samples were analyzed by the Wilcoxon matched pairs test.

For multiple comparisons among independent or paired samples, Kruskal–Wallis and Friedman tests were used, respectively, followed by Dunn’s post hoc non-parametric test.

Spearman correlation was applied to test associations between PIM mRNA and protein levels and inflammatory biological markers.

The level of statistical significance was set at *p* ≤ 0.05. Data were analyzed and graphed using the GraphPad Prism software version 9.0 (GRAPHPAD SOFTWARE, La Jolla, CA, USA).

## 5. Conclusions

The natural history of chronic inflammatory arthropathies is characterized by progressive damage to the joints, leading to disability and a deterioration in the quality of life. The drugs that are currently provided have significantly improved patients’ prognosis and, in most cases, can effectively control disease activity, but disease control fails in some cases. Therefore, there is still a need to assess novel potential biomarkers and therapeutic targets in the setting of chronic inflammatory arthropathies to focus future drug development efforts. PIM kinases are innovative targets in neoplastic diseases and, potentially, also in inflammatory diseases. This study aimed to add a few tips in the framework of the possible role of PIM kinases in chronic inflammatory arthropathies. In fact, the literature regarding the regulation of inflammation by these molecules is scarce, even more so in the rheumatological field. The main objective of this study was to assess the expression of PIM kinases in inflammatory arthritides, also related to proinflammatory cytokines. We also evaluated the therapeutic potential of targeting these molecules. In conclusion, this analysis, although preliminary, suggests the deregulation of the PIM pathway in rheumatic diseases and stimulates in-depth studies of PIM kinases’ involvement in chronic inflammatory arthropathies.

## Figures and Tables

**Table 1:** Primer sequences used in the study.

Gene	Primer Forward	Primer Reverse
*PIM1*	5′-CTG GGG AGA GCT GCC TAA TG-3′	5′-GCT CCC CTT TCC GTG ATG AA-3′
*PIM2*	5′-GCT TCT TTG GCC AAG TAG TGG-3′	5′-ACA CCC TTG TCC CAT CAA AG-3′
*PIM3*	5′-AAG ATC CTG CAG CCA GCC AA-3′	5′-CTT CAC CAC GTG CTT CAC AG-3′
*IL-17*	5′-TCT GTG ATC TGG GAG GCA AA-3′	5′-ATC TCT TGC TGG ATG GGG ACA-3′
*IL-1β*	5′-GTG GCA ATG AGG ATG ACT TGT T-3′	5′-TGG TGG TCG GAG ATT CGT AG-3′
*IL-6*	5′-TAG TGA GGA ACA AGC CAG AG-3′	5′-GCG CAG AAT GAG ATG AGT TG-3′
*TNF-α*	5′-AGC CA TGT TGT AGC AAA CC-3′	5′-ACC TGG GAG TAG ATG AGG TA-3′
*IFN-γ*	5′-TTC AGC TCT GCA TCG TTT TGG-3′	5′-TTT TCT GTC ACT CT CT TTT CC-3′
*p27*	5′-ACC TGC AAC CGA CGA TTC TT-3′	5′-GCT TCA TCA AGC AGT GAT GTA TCT-3′
*GAPDH*	5′-TGGTATCGTGGAAGGACTCATGAC-3′	5′-ATGCCAGTGAGCTTCCCGTTCAGC-3′

## Data Availability

The original contributions presented in the study are included in the article/Supplementary Materials; further inquiries can be directed to the corresponding author.

## Supplementary Materials

The supporting information can be downloaded at: https://www.mdpi.com/article/10.3390/ijms25063123/s1.
