# Supplementary material for: PIM Kinases as Potential Biomarkers and Therapeutic Targets in Inflammatory Arthritides
Source: Int J Mol Sci. 2024 Mar 8;25(6):3123. doi: 10.3390/ijms25063123 (PMC10969826; doi:10.3390/ijms25063123)
Supplement: Supplementary file 1 [file ijms-25-03123-s001.zip › ijms-2887268-supplementary.pdf]

International Journal of *Molecular Sciences*

MDPI

SUPPLEMENTARY MATERIAL

PIM kynases as potential biomarkers and therapeutic targets in inflammatory arthritides

Elisa Assirelli[1,] Jacopo Ciaffi[1], Valentina Scorcu[1], Susanna Naldi[1], Veronica Brusi[1], Luana Mancarella[1], Lucia Lisi[1], Federica Pignatti[1], Francesco Ursini[1,2*] and Simona Neri[1]

[1] Medicine and Rheumatology Unit, IRCCS Istituto Ortopedico Rizzoli, Bologna, Italy; elisa.assirelli@ior.it, jacopo.ciaffi@ior.it, susanna.naldi@ior.it; veronica.brusi@ior.it; luana.mancarella@ior.it; lucia.lisi@ior.it; federica.pignatti@ior.it; francesco.ursini@ior.it; simona.neri@ior.it

[2] Department of Biomedical and Neuromotor Sciences (DIBINEM), Alma Mater Studiorum University of Bologna, Bologna, Italy; valentina.scorcu@studio.unibo.it,

* Correspondence: francesco.ursini@ior.it

Table S1. T0 patients (naïve to bDMARD and JAKi) already treated with cDMARDs or corticosteroids. Type and duration of the treatments already ongoing at T0 together with the number of cases is indicated for each disease group. The therapy duration is expressed in months (Mean ±SD).

**Disease**	**cDMARDs**		**Cortycosteroids**	
	N. of cases	Duration	N. of cases	Duration
RA	3/23	64±80	5/23	64±62
PsA	6/24	30±33	1/24	5
axSpA	0/12	-	0/12	-

Table S2. Mean ±SD levels of inflammatory cytokines. IL-17, IL1-β, IL-6, TNF-α, and IFN-γ mRNA (n. copies/100,000 GAPDH copies) and protein (ng/ml), respectively in peripheral PBMC and in serum of RA, axSpA, PsA, and CTR samples.

Gene		RA	axSpA	PsA	CTR
IL-17	mRNA	1022±2551	169±259	102±154	817±2276
	protein	0.16±0.44	0.01±0.04	0.21±0.41	0.03±0.05
IL-1β	mRNA	19955±18284	13813±11849	19642±22590	26506±23553
	protein	0.23±0.83	0.45±1.20	0.30±1.12	0.79±2.14
IL-6	mRNA	112±87	65±43	232±192	264±303
	protein	4.51±5.15	2.25±1.74	1.83±1.45	1.79±1.87
TNF-α	mRNA	5443±4291	3652±2089	5456±4760	6908±5264
	protein	3.87±3.50	9.17±19.37	2.06±1.93	4.04±6.90
IFN-γ	mRNA	2195±2207	842±577	1802±1874	5628±6940
	protein	2.58±3.71	2.19±1.11	1.79±1.26	1.95±1.32

Table S3. Correlations between PIM kinases and inflammatory cytokines. The expression of PIM kinases (mRNAs and proteins) was correlated to the expression (mRNAs and proteins) of IL-17, IL1-β, IL-6, TNF-α, and IFN-γ inflammatory markers. Spearman correlation coefficient (a) and statistical significance (b) are reported for each group of samples (RA, axSpA, PsA, and CTR).

RA (a)	IL17 RNA	IL1beta RNA	IL6 RNA	TNFα RNA	IFNγ RNA	IFN-γ protein	IL-6 protein	IL-17 protein	TNF-α protein	IL-1β protein
PIM-1 protein	0,040491	0,080932	0,055181	-0,08216	0,01962	-0,12009	-0,16054	0,119493	0,249225	0,074174
PIM-2 protein	0,218271	0,541667	-0,09804	0,303922	0,237745	0,386959	-0,08269	0,009173	0,151703	-0,01481
PIM-3 protein	-0,52974	-0,53922	0,196078	-0,30392	-0,43137	-0,47422	0,004134	-0,12973	-0,09804	-0,13328
PIM-1 RNA	-0,41902	-0,31024	0,393624	0,22195	-0,07112	-0,07858	-0,30769	0,044878	-0,02143	0,091266
PIM-2 RNA	0,175353	0,446078	0,428922	0,460784	0,482843	0,114162	-0,26655	0,096168	0,435714	0,304869
PIM-3 RNA	-0,31718	0,217045	0,05886	0,403434	0,290619	-0,15568	-0,15027	-0,25645	-0,30714	-0,26021

RA (b)	IL17 RNA	IL1beta RNA	IL6 RNA	TNFα RNA	IFNγ RNA	IFN-γ protein	IL-6 protein	IL-17 protein	TNF-α protein	IL-1β protein
PIM-1 protein	0,8773782	0,7574884	0,8333976	0,7539199	0,9404219	0,6243483	0,5245427	0,6367367	0,3186114	0,7628179
PIM-2 protein	0,399999	0,0247148	0,7081513	0,2356358	0,3581714	0,1016933	0,744283	0,9711841	0,5479007	0,9520199
PIM-3 protein	0,02873954	0,02550368	0,450705	0,2356358	0,08383074	0,04023485	0,9870099	0,6079122	0,6987425	0,5864798
PIM-1 RNA	0,09410363	0,22555	0,1180008	0,3919032	0,7861985	0,7723765	0,2645737	0,8738164	0,9395779	0,736754
PIM-2 RNA	0,5008376	0,07269184	0,08580102	0,06267762	0,04962159	0,6737636	0,3369021	0,7331517	0,1044934	0,2509064
PIM-3 RNA	0,2147957	0,4027174	0,8224517	0,1083133	0,2577924	0,5648041	0,5929616	0,3562084	0,2654727	0,3304032

axSpA (a)	IL17 RNA	IL1beta RNA	IL6 RNA	TNFα RNA	IFNγ RNA	IFN-γ protein	IL-6 protein	IL-17 protein	TNF-α protein	IL-1β protein
PIM-1 protein	0,166667	0,233333	-0,48333	0,633333	-0,5	0,1	0,566667	0	-0,38657	-0,42829
PIM-2 protein	0	0,25	-0,28571	0,607143	-0,17857	0,166667	-0,02381	-0,24744	0,467074	-0,45363
PIM-3 protein	0,392857	-0,5	0	0,392857	-0,67857	0,119048	0,285714	0,57735	-0,29941	-0,06873
PIM-1 RNA	-0,02381	0,690476	-0,59524	0,880952	0,238095		0		0	-0,7746
PIM-2 RNA	-0,1	0,75	-0,3	0,333333	0,733333	-0,6	-0,6		0,8	-0,70711
PIM-3 RNA	0,833333	0,566667	-0,51667	-0,21667	0,466667	-0,4	-0,5		0,7	-0,35355

axSpA (b)	IL17 RNA	IL1beta RNA	IL6 RNA	TNFα RNA	IFNγ RNA	IFN-γ protein	IL-6 protein	IL-17 protein	TNF-α protein	IL-1β protein
PIM-1 protein	0,6777447	0,5517086	0,1938	0,076036	0,177662	0,809981	0,120574		0,3125	0,249917
PIM-2 protein		0,5948412	0,555952	0,166667	0,713095	0,703323	0,976786	0,536409	0,243056	0,26746
PIM-3 protein	0,3956349	0,2666667		0,395635	0,109524	0,793006	0,500794	0,151141	0,461806	0,881994
PIM-1 RNA	0,9767857	0,06939484	0,132292	0,007242	0,582143					0,333333
PIM-2 RNA	0,8099813	0,02549052	0,436624	0,385323	0,031123	0,35	0,35		0,133333	0,233333
PIM-3 RNA	0,008267196	0,1205743	0,1618	0,580941	0,212522	0,516667	0,45		0,233333	0,516667

**PsA (a)**	IL17 RNA	IL1beta RNA	IL6 RNA	TNFα RNA	IFNγ RNA	IFN-γ protein	IL-6 protein	IL-17 protein	TNF-α protein	IL-1β protein
PIM-1 protein	-0,04644	-0,44892	-0,29205	-0,3459	-0,69363	-0,12569	-0,05623	-0,61054	-0,22712	-0,168
PIM-2 protein	0,184727	-0,14551	0,0258	-0,14662	-0,4902	0,017621	-0,16079	-0,44077	-0,02643	-0,11993
PIM-3 protein	-0,01548	-0,26316	0,027864	-0,02994	-0,14951	-0,23568	0,026432	-0,15247	-0,30176	-0,1476
PIM-1 RNA	0,19618	0,437791	0,254001	0,378099	0,586144	0,198206	0,252263	0,408248	0,214286	0,174608
PIM-2 RNA	0,023748	0,409912	0,284977	0,466942	0,388719	0,306319	0,846881	0	0,428571	0,058026
PIM-3 RNA	0,230372	0,342975	0,022727	0,312145	0,384049	0,612638	0,180188	-0,40825	-0,21429	0,058203

**PsA (b)**	IL17 RNA	IL1beta RNA	IL6 RNA	TNFα RNA	IFNγ RNA	IFN-γ protein	IL-6 protein	IL-17 protein	TNF-α protein	IL-1β protein
PIM-1 protein	0,8548139	0,06165736	0,2396018	0,1597231	0,002014144	0,6685461	0,8485844	0,02039395	0,4348778	0,519232
PIM-2 protein	0,4630613	0,5645405	0,9190605	0,5615478	0,04575998	0,9523236	0,5829124	0,1146868	0,9285316	0,6466157
PIM-3 protein	0,951388	0,2913924	0,9126079	0,9061123	0,5668308	0,4172783	0,9285316	0,6028207	0,2943982	0,5718447
PIM-1 RNA	0,4352767	0,06921345	0,309117	0,1218478	0,01340807	0,661508	0,5948412	0,3535714	0,661508	0,6321418
PIM-2 RNA	0,9254795	0,0911348	0,2517035	0,05074582	0,1230659	0,497619	0,02380952		0,3535714	0,8651444
PIM-3 RNA	0,3577433	0,163514	0,9286748	0,2072938	0,128027	0,1666667	0,7130952	0,3535714	0,661508	0,8651444

**CTR (a)**	IL17 RNA	IL1beta RNA	IL6 RNA	TNFα RNA	IFNγ RNA	IFN-γ protein	IL-6 protein	IL-17 protein	TNF-α protein	IL-1β protein
PIM-1 protein	-0,153846	-0,237762	-0,202797	0,069930	0,163636	0,581437	0,517618	0,129550	-0,091068	0,599106
PIM-2 protein	0,097902	0,034965	0,286713	0,328671	0,518182	0,672505	0,588043	0,194325	0,112084	0,703118
PIM-3 protein	-0,272727	-0,615385	-0,139860	-0,314685	-0,190909	0,658495	0,274655	0,000000	-0,178634	0,391083
PIM-1 RNA	0,048951	0,734266	0,643357	0,762238	0,454545	-0,290718	0,274655	0,518200	-0,126095	-0,054086
PIM-2 RNA	-0,073555	0,784590	0,588442	0,697024	0,527273	-0,371930	0,029983	0,259554	-0,105263	-0,166710
PIM-3 RNA	0,650350	0,524476	0,230769	0,475524	0,527273	0,091068	0,359164	0,259100	0,444834	0,436848

**CTR (b)**	IL17 RNA	IL1beta RNA	IL6 RNA	TNFα RNA	IFNγ RNA	IFN-γ protein	IL-6 protein	IL-17 protein	TNF-α protein	IL-1β protein
PIM-1 protein	0,633091	0,456801	0,527302	0,829024	0,630685	0,047374	0,084773	0,688213	0,778346	0,039533
PIM-2 protein	0,762122	0,914093	0,366251	0,296904	0,102492	0,016569	0,044325	0,545053	0,728736	0,010747
PIM-3 protein	0,391097	0,033170	0,664633	0,319139	0,573913	0,019892	0,387623	1	0,578567	0,208728
PIM-1 RNA	0,879919	0,006543	0,024003	0,003950	0,160145	0,359293	0,387623	0,084362	0,696174	0,867416
PIM-2 RNA	0,820285	0,002512	0,044145	0,011761	0,095565	0,233853	0,926303	0,41526	0,744743	0,604564
PIM-3 RNA	0,022034	0,080019	0,470532	0,118176	0,095565	0,778346	0,251553	0,416106	0,147339	0,155619
